# A cross-sectional analysis of the geographic distribution and causes of maternal mortality in South Africa: 2002–2006

**DOI:** 10.1186/s12889-015-1597-5

**Published:** 2015-03-19

**Authors:** Nolunkcwe J Bomela

**Affiliations:** Department of Research Management, Nelson Mandela Metropolitan University, University Way, Summerstrand, Port Elizabeth, Eastern Cape 6031 South Africa

**Keywords:** Geographic, Provincial, Maternal mortality, Direct causes, Indirect causes

## Abstract

**Background:**

Major changes in health policy, health service delivery, specific protocols, guidelines and recommendations for the management of common causes of maternal death have been developed in South Africa since the advent of the current democratic government. However, maternal mortality ratio remains high. The scientific community has conducted numerous studies on maternal mortality in South Africa; save for an analysis of the causes of maternal deaths, stratified by province. This study examines the geographic distribution of maternal causes of death in South Africa.

**Methods:**

A pooled cross-sectional dataset for the years 2002–2006 retrieved from the vital registration database of Statistics South Africa was used to analyse maternal causes of death. About 8773 maternal deaths between 10–55 years were analysed using frequency tables, cross-tabulations and logistic regression. Maternal mortality ratios (MMR), odds ratios (OR) and 95% confidence intervals (CI) were used to analyse provincial disparities.

**Results:**

MMR was highest in the Free State (286/100 000) and lowest in the Western Cape (87/100 000). Tuberculosis (10.4%) was the leading single indirect cause of maternal deaths while hypertensive disorders (9.1%) were the leading direct cause of death. KwaZulu-Natal women had a significantly higher risk of dying from sepsis (aOR=3.1,95% CI=1.2-7.9). North West women had the lowest risk of dying from hypertensive disorders (aOR=0.4,95% CI=0.2-0.7). The risk of dying from complications of labour was lowest for Gauteng women (aOR=0.4,95% CI=0.1-0.9). The 30–34 years age group had a significantly high risk (aOR=2.5,95% CI=1.6-4.0) of dying from abortion while the 25–29 years age group had a significantly higher risk of dying from maternal infectious diseases (aOR=2.3,95% CI=1.3-3.9). The 40–44 years age group had a significantly higher risk of dying from haemorrhage (aOR=2.3,95% CI=1.3-3.9 and the 45+ age group from other maternal diseases (aOR=3.3,95% CI=1.2-8.5) and miscellaneous direct causes (aOR=4.1,95% CI=1.7-9.9) respectively.

**Conclusions:**

The study shows great variations in the distribution and causes of maternal deaths by age and provincial level. Poorer provinces had lower MMR than the better off provinces. The provincial variations in the leading causes of death indicate the importance of targeted interventions at sub-national level.

**Electronic supplementary material:**

The online version of this article (doi:10.1186/s12889-015-1597-5) contains supplementary material, which is available to authorized users.

## Background

The endorsement of the Millennium Declaration by all United Nations (UN) member states in the year 2000 served as a catalyst for substantial improvements in amongst others, maternal health, defined by Millennium Development Goal 5 (MDG5), which includes the specific target of reducing maternal mortality by 75% between 1990 and 2015 [1]. Governments worldwide began defining their own policies and goals for maternal mortality reduction accordingly. South Africa also embarked on its own plan of action. Besides, South Africa had already established in 1997 the National Committee on Confidential Enquiries into Maternal Deaths (NCCEMD). In the same year, maternal death became a notifiable condition [2]. Major changes in health policy, and health service delivery were introduced. Specific protocols, guidelines and recommendations for the management of common causes of maternal death were developed. Despite these endeavours the maternal mortality ratio (MMR) remains high in South Africa and there are substantial variations in provincial maternal mortality [2-6].

Narrowing the provincial gap in MMR is a difficult challenge faced by the South African government as it attempts to reduce maternal deaths. Moreover, the estimation of the MMR in South Africa varies widely by source and method of estimation and is highly contentious, owing to estimates from different data sources and estimation procedures that vary widely. A range of estimates have been produced based on data from various sources all of which have limitations which render estimates produced debatable. These estimates are often inconsistent with one another, which partly reflects the type and quality of data sources and partly the methodology used in deriving them. This has created uncertainty for policymakers with regard to the actual level and trends of maternal mortality in the country. Other estimates are derived from modelling a combination of these data sources, for example by, WHO, UNICEF, UNFPA and The World Bank [7]. The most recent estimations of the population based MMR for South Africa vary between 150/100000 live births (1998, Demographic Health Survey); 181-382/100000 live births (Graham and Newell); 240-400/100000 live births (UN estimates); 578/100000 live births (2001 South Africa Census estimates) and 764/100000 (2007 South Africa Community Survey estimates) [1,3].

The Confidential Enquiries system ensures through a specially developed maternal death notification that all institutional maternal deaths are directly reported to the Department of Health. However, most maternal deaths occurring outside of health institutions are not reported to the NCCEMD. Some of these deaths are registered and captured annually by the Department of Home Affairs (DHA), (including late registrations) and subsequently analysed by Statistics South Africa (StatsSA). In the fourth NCCEMD report published in 2008 the ‘big five’ maternal causes of death have remained the same since the first report was published in 1998, mainly – non-pregnancy related infections including AIDS, complications of hypertension, antepartum and postpartum haemorrhage, early pregnancy losses related to septic abortions and pre-existing maternal disease [3,4]. Besides, non-pregnancy related infections have been the leading cause of maternal deaths since 1999. Available statistics from vital registration data from StatsSA indicate that 12 429 women died of pregnancy related causes between 1999 and 2006 in South Africa [8].

Reducing maternal deaths in South Africa under the same policies is a great challenge. This is partly due to the fact that health policy is directed from a national perspective, and provincial and local governments have to adapt it to the specific needs of their individual provinces. Conversely, this demands not only an all-inclusive understanding of the causes but also, more notably, an understanding of how the different causes are distributed in the various provinces that have different physiognomies. Consequently, provinces have to develop specific protocols that can address maternal mortality within the provinces. The development of the protocols is not always in sync with the specific causes of maternal death in each province. Reliable information about provincial maternal mortality in South Africa is essential in order to identify where the risk is highest as well as where the numbers of deaths are the largest within the country. This information is essential for mobilization of resources and implementation of specific policies to reduce MMR. In China, for example, in order to monitor local maternal mortality levels and to provide scientific evidence for mother and child health (MCH) interventions suitable to the local situation, provincial maternal mortality surveillance systems (PMMSS) have been set up in nearly all the provinces [9].

Differences in maternal mortality within countries are found everywhere and are extensive [10]. Women in eastern Uganda were reported to have a high risk of death compared with the central and western regions [11]. In India, between the years 2007 and 2009 the MMR in four states (Andhra Pradesh, Karnataka, Kerala, and Tamil Nadu) ranged from 81/100 000 and 178/100 livebirths [12]. Additionally, within India, there is marked variation in healthcare access between regions and in socioeconomic factors. The rural areas of poorer states have a higher proportion of maternal deaths than the richer states [12,13]. Gan et al. [9] found in China that provinces in remote regions (low socioeconomic level provinces) had the highest risk of maternal mortality, followed by provinces in the inland regions (moderate socioeconomic level provinces) and then the coastal regions (high socioeconomic level provinces).

In South Africa, several studies have been conducted on maternal mortality with the single aim of understanding and optimising health outcomes for pregnant women [14-16]. However, only one study by Garenne et al. [14] attempted to analyse the maternal deaths with respect to their geographical distribution using the 2001 South African Census. Garenne et al. [14] reported the differentials in MMR by residence, province, race, language, education, and income and wealth index. Risk factors on maternal mortality at household and provincial level were also reported. However, the study did not report on maternal mortality causes as this information is not captured by the Census, but it did give a description of maternal mortality levels in the provinces. Hence, an analysis of the causes of maternal deaths, stratified by province is essential for targeted implementation of the specific protocols, guidelines and recommendations by the NCCEMD.

## Methods

### Source of data

A pooled cross-sectional data set from the 2002–2006 Causes of Death data sets from StatsSA was used to assess and analyse the distribution of maternal deaths in South Africa. This included vital registration data on the recorded/estimated number of maternal deaths and live births for the period under study. In South Africa, deaths are registered with the DHA. The DHA captures the information available on the death notification form (DNF) in order to be able to issue a death certificate. All causes of death and conditions leading to death are entered on the DNF– the immediate, second, third, fourth, other condition and the underlying cause. An *underlying cause* of death is defined as the disease or injury, which initiated the train of morbid events leading directly to death, or the circumstances of the accident or violence, which produced the fatal injury [17]. Each deceased person in South Africa is allocated one underlying cause of death on the death certificate. At the end of each year, the DNFs are photocopied and sent to StatsSA for further analysis. At StatsSA, professional nurses/nosologists, who have also been trained in allocation of ICD-10 codes to causes of death, code and capture the codes [18]. Upon completion of each year, statisticians and demographers analyse the causes of death and publish the relevant reports. It should be noted however, that, South Africa has an unacceptably high proportion of DNFs that contain errors and these errors inadvertently affect the accuracy of the cause of death coding. Another limitation of the data is the probable under-registration of deaths, particularly in rural areas and among children. This leads to lower estimates of the total number of deaths that have occurred in the country and may lead to under-estimation of some causes of death [18,19].

It is important to note that the number live births and deaths processed by Stats SA is always higher than the number of live births and deaths recorded on the National Population register (NPR) for the same period. The NPR includes only South African citizens and permanent residents whose births are registered while StatsSA processes all death notification forms regardless of civil status [17]. Data was converted from ASCII format to STATISTICA 10 for the analyses. All cases with the variable pregnant were selected from the causes of death data sets and new files were created and merged to form one file. Unfortunately, the data from StatsSA did not include other central and relevant variables to allow for the analysis of the influence of social factors on maternal mortality.

### Statistical analysis

Univariate analyses of all maternal deaths for each year and for all the independent variables relevant to the study were carried out. Thereafter, bivariate analyses (of pooled data for the five years under study in order to create a more usable dataset for the logistic regression analysis) were carried out. Variables included were maternal age, province of death, place of death and causes of death. The final causes of death were grouped into 19 categories due to the substantial disparities in maternal deaths in South Africa. Each of the causes classified under the 19 categories was dichotomised and cross-tabulated with the independent variables to analyse how the causes differ in the different groups. Variables that indicated a significant association in the bivariate analyses were included in the logistic regression analysis. All those variables that had a significance level of 0.05 for the Wald statistic were used in the logistic regression. The 10–19 years age group was taken as a reference category because it had the lowest number of deaths during this period. The Western Cape was taken as a reference group because it had the lowest MMR and the health care facility was used because most deaths occurred at a health care facility. Hosmer and Lemeshow tests were performed to evaluate the model fitting for all logistic regressions, all models had low chi-square values and p values for all the models were > 0.05. The leverage and the standardized residual statistics of each observation were performed and no influential outliers were observed.

Maternal and late maternal deaths were defined as follows: “*Maternal death*: the death of a woman while pregnant or within 42 days of termination of pregnancy, irrespective of the duration and site of the pregnancy, from any cause related to or aggravated by the pregnancy or its management but not from accidental or incidental causes. A *late maternal death* is the death of a woman from a direct or indirect obstetric cause more than 42 days but less than one year after termination” [20]. “*Direct obstetric deaths:* those resulting from obstetric complications of the pregnant state (pregnancy, labour and puerperium), from interventions, omissions, incorrect treatment, or a chain of events resulting from any of the above. *Indirect obstetric deaths:* those resulting from previous existing disease or disease that developed during pregnancy and which was not due to direct obstetric causes, but which was aggravated by physiologic effects of pregnancy” [18].

## Results

A total of 8 773 maternal deaths between 10-50+ years old were analysed. Considering that the probability of dying during pregnancy is small, the data for the five years was combined to get a more balanced MMR. The MMRs reported in this study (formula below) were calculated by dividing the recorded/estimated number of maternal deaths by the total recorded/estimated number of live births (retrieved from StatsSA database) between 2002 and 2006 and multiplying the result by 100,000 [20,21].

$$ \mathrm{Maternal}\;\mathrm{mortality}\;\mathrm{ratio}\;\left(\mathrm{M}\mathrm{M}\mathrm{R}\right)=\frac{\mathrm{Number}\;\mathrm{of}\;\mathrm{maternal}\;\mathrm{deaths}\;\mathrm{in}\;\mathrm{a}\;\mathrm{given}\;\mathrm{year}\;\mathrm{a}\mathrm{nd}\;\mathrm{a}\mathrm{rea}}{\mathrm{Number}\;\mathrm{of}\;\mathrm{live}\;\mathrm{births}\kern0.24em \mathrm{in}\kern0.24em \mathrm{the}\;\mathrm{s}\mathrm{a}\mathrm{me}\;\mathrm{year}\;\mathrm{a}\mathrm{nd}\;\mathrm{a}\mathrm{rea}}\times 100,000\;\mathrm{live}\mathrm{s}\;\mathrm{births} $$

**Figure 1:**
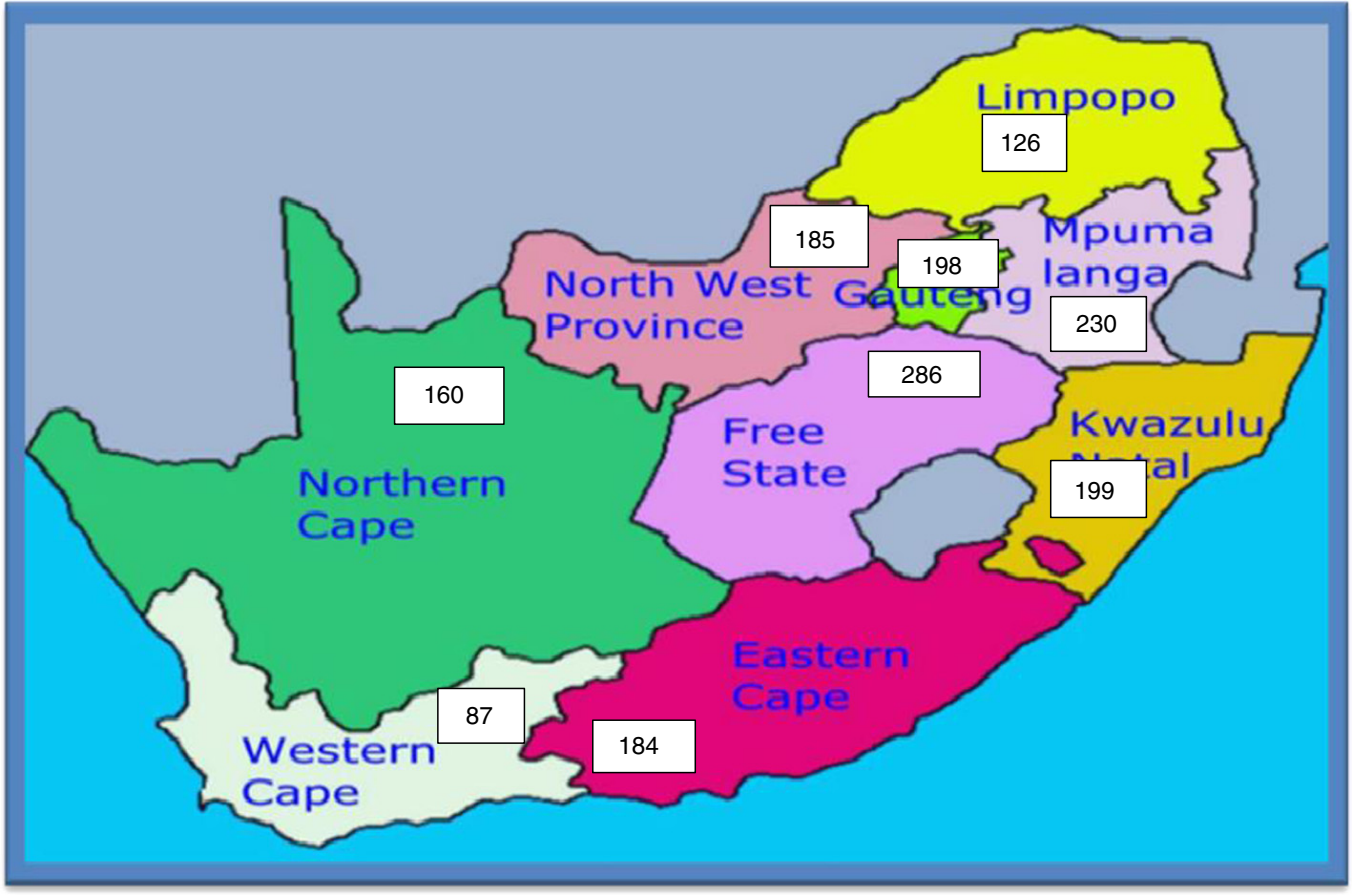
**Maternal mortality ratio per province per 100 000 births.** Source: computed from 2002–2006 maternal deaths and live births data. Statistics South Africa.

**Figure 2:**
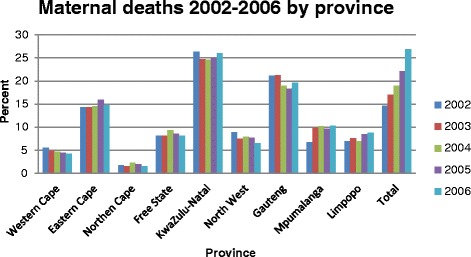
**Maternal deaths 2002–2006 by province.**

Provincial level estimates indicate that the Free State had the highest MMR of 286/100 000 live births, higher than the national average of 183/100 000 live births during this period while the Western Cape had the lowest MMR (87/100 000 live births) and was the only province that had a steady decline in maternal deaths during this period (see Figures 1 and 2). The overall MMR (183/100 000 live births) for the period under review according to data from StatsSA is substantially lower than the 2007 MMR (299/100 000 live births) computed from civil registration and vital statistics systems [1] and the 2007 South Africa Community Survey MMR (764/100 000 live births) computed by Udjo et al. [22] indicating a significant increase in maternal mortality ratio after 2006. A substantial annual increase in maternal deaths between 2002 and 2006 occurred in South Africa as depicted in Figure 2. Noteworthy, slightly more than a quarter of all the deaths in this period occurred in 2006 alone. The distribution of maternal deaths and related factors also varies markedly among different provinces within the country (Table 1). The burden of maternal mortality is greatest in the most populous provinces viz. KwaZulu-Natal (KZN), Gauteng (GT) and Eastern Cape (EC).

**Table 1:** **Socio-demographic characteristics of women who died from pregnancy-related causes in South Africa (2002–2006)**

**Variable**	**Number**	**Valid (%)**
**Age group (years)**		
10-14	30	0.3
15-19	710	8.1
20-24	1691	19.3
25-29	2239	25.5
30-34	2086	23.8
35-39	1185	13.5
40-44	536	6.1
45-49	215	2.5
50+	81	0.9
**Total**	**8773**	**100.0**
**Place of Death**		
Health care facility	6469	73.8
Home	1208	13.7
Other	1096	12.5
**Total**	**8773**	**100.0**
**Province of Death**		
Western Cape	415	4.7
Eastern Cape	1302	14.8
Northern Cape	158	1.8
Free State	744	8.5
KwaZulu-Natal	2221	25.3
North West	665	7.6
Gauteng	1731	19.7
Mpumalanga	839	9.6
Limpopo	698	7.9
**Total**	**8773**	**100.0**

Table 1 shows the distribution of maternal deaths by age group, place of death and province of death. The mean age at death was 29.3 years, (SD 7.4). The highest number of deaths occurred in the 25 to 29 years age group. Moreover, slightly more than half the maternal deaths occurred in women below the age of 30 during this period. Significantly, throughout this period not only were the deaths concentrated in these age groups but that they were also increasing annually^a^ Almost three quarters (73.8%) of the deaths occurred at a health care facility, 13.7% deaths occurred at home and 12.5% at an unspecified place of death.

**Figure 3:**
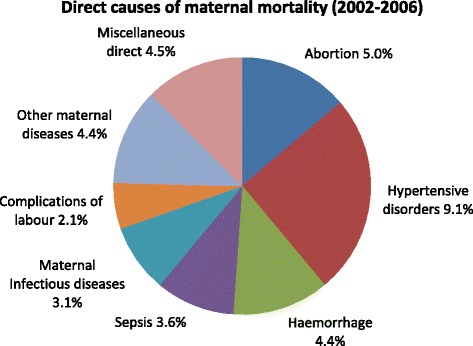
**Direct causes of maternal mortality (2002–2006).**

**Figure 4:**
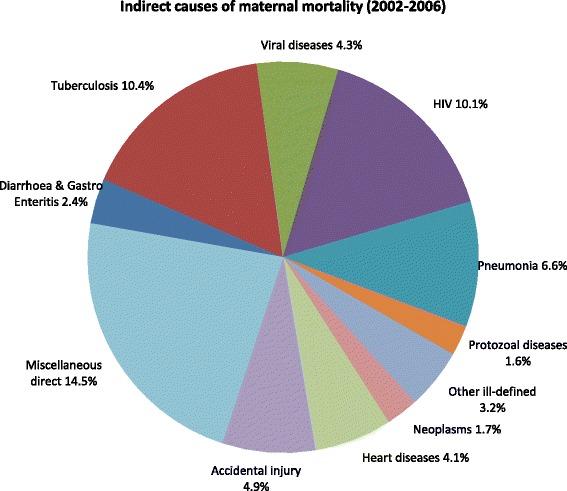
**Indirect causes of maternal mortality (2002–2006).**

Figures 3 and 4 show the distribution of maternal causes of death. Hypertensive disorders were the most common direct cause of maternal deaths (9.1%). Among the indirect causes of maternal deaths, miscellaneous indirect causes accounted for 14.5% of deaths, followed by tuberculosis (10.4%) and HIV and related causes (10.1%).

(see Additional file 1: Tables S1 and S2) show the variations in the indirect and direct causes of death by age group, province of death and place of death.

Diarrhoea and gastro-enteritis, tuberculosis, viral diseases, HIV and related causes, pneumonia, and protozoal diseases were highest in the 25–29 years age group. In contrast, other ill-defined causes, heart diseases and miscellaneous indirect causes mainly occurred in the 30–34 years age group. Moreover, women in the 20–39 years age group seem to be more vulnerable to heart diseases than the women above 40 years and those below 20 years. Although neoplasms were highest in the 45–49 years age group an increase with age in deaths from neoplasms was evident, while accidental injury was highest in the 20–24 years age group followed closely by the 10–19 years age group. No deaths from protozoal diseases occurred in the 45+ age group.

Maternal mortality was highest for women who died in KwaZulu-Natal in eight indirect causes while Gauteng had the highest number of deaths in three indirect causes. No deaths from neoplasms were reported in the Northern Cape. The majority of deaths from indirect causes occurred in a health care facility except for a larger number of deaths from other ill-defined causes which occurred at home and accidental injury which occurred at an unspecified place of death respectively.

Hypertensive disorders, sepsis, maternal infectious diseases and other maternal diseases were highest in the 25–29 years age group. Conversely, abortion, haemorrhage, complications of labour and miscellaneous direct causes occurred mainly in the 30–34 years age group. No deaths from sepsis were reported in the 45+ age group. KwaZulu-Natal had the highest number of deaths from six direct causes while Gauteng had the highest number of deaths in two direct causes. The majority of maternal deaths from direct causes occurred in a health care facility.

(see Additional file 2: Table S3) shows the adjusted odds ratios for indirect causes. The 35–39 years age group had a significantly reduced risk of dying from diarrhoea & gastro enteritis (aOR = 0.4, 95% CI = 0.1-0.8) compared to other women. Women in the 20–44 years age group had a significantly reduced risk of dying from tuberculosis. The risk of dying from viral diseases was significantly lower for the 25–29 years age group (aOR = 0.2, 95% CI = 0.2-0.8) and the 35–39 years age group (aOR = 0.5, 95% CI = 0.3-0.9). The 20–39 years age group had a significantly lower risk of dying from HIV and the 30–34 years old women had the lowest risk (aOR = 0.4, 95% CI = 0.3-0.7) compared to other women including the reference group. Women in the 35–39 years age group had a significantly reduced risk of dying from protozoal diseases (aOR = 0.3, 95% CI = 0.1-1.0). The risk of dying from other miscellaneous indirect causes was significantly high for women below 34 years old. The risk of dying from neoplasms was significantly lowest for the 40–44 years old women (aOR = 0.3, 95% CI = 0.1-0.7). Conversely, it was high but not significant for women between 20 and 34 years old. Women in the 25–29 years age group had a significantly high risk of dying from heart diseases (aOR = 2.0, 95% CI = 1.2-3.1). No significant differences were observed regarding pneumonia, other ill-defined diseases and accidental injury. However, the risk of dying from accidental injury was high to very high for all the age groups compared to the reference group.

Women from the KwaZulu-Natal (aOR = 0.3, 95% CI = 0.1-0.9) North West (aOR = 0.3, 95% CI = 0.1-0.9), Mpumalanga (aOR = 0.2, 95% CI = 0.0-0.6) and Limpopo (aOR = 0.2, 95% CI = 0.0-0.6) had a significantly reduced risk of dying from diarrhoea & gastro-enteritis. The risk of dying from tuberculosis was significantly low for KwaZulu-Natal women (aOR = 0.6, 95% CI = 0.4-0.9) compared to other women. Gauteng women had a lower risk (aOR = 0.5, 95% CI = 0.3-1.01) of dying from viral diseases while Limpopo women had a high (aOR = 1.9, 95% CI = 0.9-4.2) but not significant risk. The risk of dying from HIV was significantly high for all the provinces and the risk was highest in Limpopo (aOR = 2.6, 95% CI = 1.7-4.0) although not significant compared to women from other provinces. All eight provinces had a significantly reduced risk of dying from pneumonia. Free State (aOR = 0.2, 95% CI = 0.0-0.7) and KwaZulu-Natal women (aOR = 0.3, 95% CI = 0.0-1.0) had a reduced risk of dying from protozoal diseases. The risk of dying from accidental injury was very high for women from Mpumalanga (aOR = 4.1, 95% CI = 2.2-7.6) and lowest for Northern Cape women (aOR = 0.7, 95% CI = 0.3-1.4) but was not significant. However, a significantly high risk was observed for women from Eastern Cape, Free State, KwaZulu-Natal, North West and higher for Gauteng women (aOR = 2.5, 95% CI = 1.6-4.1). No significant differences were observed regarding other ill-defined diseases and heart diseases. However, the risk of dying from other ill-defined diseases was high but not significant for Mpumalanga women (aOR = 1.7, 95% CI = 0.7-3.8) while the risk of dying from other heart diseases was high but not significant for Northern Cape women (aOR = 1.5, 95% CI = 0.5-4.1) and North West women (aOR = 1.5, 95% CI = 0.7-2.9) compared to women from other provinces.

Women who died at home had a significantly reduced risk of dying from diarrhoea & gastro enteritis (aOR = 0.5, 95% CI = 0.3-0.7) and pneumonia (aOR = 0.6, 95% CI = 0.5-0.8). Conversely, they had a significantly very high risk of dying from protozoal diseases (aOR = 8.6, 95% CI = 2.7-27.3) a high risk of dying from neoplasms (aOR = 1.6, 95% CI = 0.9-2.7) and viral diseases (aOR = 1.7, 95% CI = 1.1-3.0). Deaths from tuberculosis (aOR = 1.7, 95% CI = 1.3-2.1) and HIV (aOR = 2.3, 95% CI = 1.8-2.3) were high but not significant. No significant differences were observed regarding other indirect diseases, other ill-defined, heart diseases and accidental injury for deaths that occurred at home. Women who died elsewhere had a significantly high risk of dying from tuberculosis (aOR = 1.6, 95% CI = 1.2-2.0), pneumonia (aOR = 1.3, 95% CI = 1.0-1.8) and a significantly high risk for protozoal diseases (aOR = 1.9, 95% CI = 1.0-3.8) compared to those who died at a health care facility.

(see Additional file 2: Table S4) shows the adjusted odds ratios for direct causes. Women below 39 years had a significantly high risk of dying from abortion and a significantly higher risk was noted in the 30–34 years age group (aOR = 2.5, 95% CI = 1.6-4.0) compared to other women. A significantly lower risk of dying from hypertensive disorders was observed in the 20–24 years age group (aOR = 0.6, 95% CI = 0.4-0.8), the 40–44 years age group (aOR = 0.6, 95% CI = 0.4-0.6) and a significantly reduced risk in the 45+ age group (aOR = 0.2, 95% CI = 0.0-0.7). Compared to the reference group, the risk of dying from haemorrhage was significantly high in the 35–44 years age group and highest in the 40–44 years age group (aOR = 2.3, 95% CI = 1.3-3.9). The 30–34 years age group had a significantly reduced risk of dying from sepsis (aOR = 0.6, 95% CI = 0.4-0.9) compared to other women. Deaths from maternal infectious diseases were significantly high in the 25–34 years age group and highest in the 25–29 years age group (aOR = 2.3, 95% CI = 1.3-3.9). No significant differences were observed between the age groups in relation to deaths from complications of labour. The 45+ years old age group had a significantly high risk of dying from other maternal diseases (aOR = 3.3, 95% CI = 1.2-8.5) and miscellaneous direct diseases (aOR = 4.1, 95% CI = 1.7-9.9) compared to other women.

There were no significant differences observed between the provinces in relation to abortion, maternal infectious diseases, other maternal diseases, complications of labour and other miscellaneous direct causes of death. However, the risk of dying from other maternal diseases was moderately high for women from the North West although not significant (aOR = 1.8, 95% CI = 0.9-3.7). Women from the Northern Cape had a moderate but not significant risk of dying from maternal infectious diseases (aOR = 1.6, 95% CI = 0.5-4.7), while Gauteng women had a lower (aOR = 0.5, 95% CI = 0.2-1.2) but not significant risk compared to women from other provinces. The risk of dying from hypertensive disorders was significantly reduced for women from KwaZulu-Natal (aOR = 0.6, 95% CI = 0.4-1.0), Mpumalanga (aOR = 0.5, 95% CI = 0.3-0.8) and lowest for women from the North West (aOR = 0.4, 95% CI = 0.2-0.7) compared to other provinces. Women from the Eastern Cape had a significantly high risk of dying from haemorrhage (aOR = 2.0, 95% CI = 0.9-4.2). However, all other provinces had a high risk although not significant compared to the Western Cape. The risk of dying from sepsis was significantly high for women from KwaZulu-Natal (aOR = 3.1, 95% CI = 1.2-7.9) the Eastern Cape (aOR = 2.8, 95% CI = 1.1-7.2), and the North West (aOR = 2.7, 95% CI = 1.0-7.3). Limpopo (aOR = 2.4, 95% CI = 0.9-6.5) and Free State (aOR = 2.1, 95% CI = 0.8-5.7) women had a high but not significant risk of dying from sepsis.

There were no significant differences observed between the health care facilities, home and other place of death regarding abortion, hypertensive disorders, haemorrhage, maternal infectious diseases, and complications of labour and miscellaneous direct causes. The risk of dying from other maternal diseases (aOR = 1.4, 95% CI = 1.0-1.9) and sepsis (aOR = 1.5, 95% CI = 1.0-2.1) was significantly high for women who died at home while the risk of dying from sepsis was significantly low for women who died elsewhere (aOR = 0.6, 95% CI = 0.4-0.9) compared to those who died at a health care facility. Women who died elsewhere had a significantly reduced risk of dying from maternal infectious diseases (aOR = 0.5, 95% CI = 0.3-0.9) but a significantly high risk of dying from complications of labour (aOR = 1.5, 95% CI = 1.0-2.3) than the reference group.

## Discussion

The study revealed significant geographic variations in maternal deaths and causes of death, which suggests the importance of maternal health interventions at subnational level. This geographical variation may be related to the different socio-economic, demographic and environmental features in the provinces. Similar findings were reported in China and Uganda [11,21]. The WC, NW and GT showed a steady decline in maternal mortality deaths during this period, the deaths in other provinces either increased or remained constant. As expected, the most populous provinces KZN, EC and GT had the highest numbers of deaths. Other studies conducted in South Africa also found that these three provinces presented with the highest numbers of maternal deaths [4,7,22]. This could be due to a number of reasons, a combination of better reporting, an actual increase in deaths and poor health care services. In China, it has been reported that underdeveloped, remote areas and rural areas had higher MMRs than developed, coastal and urban areas [23]. In South Africa the circumstances are somewhat different. The gap in the MMR between the four coastal provinces (NC, WC, EC and KZN) is wide and there are vast differences in their economic levels. Amongst these provinces the Western Cape, had the lowest MMR. Similar findings have been reported by Garenne et al. and all the NCCEMD reports [2-6,14]. Living standards and socio-economic conditions in the Western Cape are relatively high by national standards and the province fares well in providing access to healthcare whilst the other three provinces are lagging behind in socio-economic development compared to the Western Cape [24]. The MMR in the five inland provinces also varies widely. Gauteng, the economic hub of South Africa, is mainly driven by the finance and business services sub-sector; it is the most similar province to the Western Cape by socio-economic development standards but had more than double the Western Cape MMR. The Free State province considered to be the “breadbasket” of the country with 3.2 million hectares of cultivated land had the highest MMR in the country higher than the national average at the time [24].

The differences in MMR described here indicate that maternal mortality cannot simply be explained by variations in economic development, societal factors heavily influence maternal mortality levels and outcomes. These differences also highlight the importance of the health care system in the delivery of quality care. Ronsmans et al. [25] reported that physical access to obstetric care, differences in the uptake of antenatal and delivery services were some of the reasons for elevated MMR in the urban and rural areas and amongst the poor and rich of Afghanistan and Peru respectively. In South Africa various reports by the NCCEMD indicate that women in the poorer and rural provinces do not have adequate access to health care and specifically obstetric care. High illiteracy, poor quality of health care service, poor transport facilities have been cited in the NCCEMD reports as some of the reasons that negatively influence maternal health.

National level figures indicate that pregnancy, childbirth and puerperium related deaths (direct) were the leading causes of death throughout this period [3,6]. However, national figures invariably mask substantial internal variation, be they geographical, economic, social or rural–urban. In this study, the leading causes of death at provincial level varied widely. KwaZulu-Natal had the highest percentage of both direct (23%) and indirect (27%) causes, followed by Gauteng with 20% indirect and 19% direct causes of death. Further investigations are necessary in order to identify the reasons for the high percentages in these provinces besides the populous nature of these two provinces.

Nationally, tuberculosis was the leading indirect cause of maternal deaths while hypertensive disorders were the leading direct cause. Tuberculosis has been identified as the most opportunistic infection causing maternal mortality in South Africa pre and post-partum [2-6]. Burton [26] suggests that midwives should perform symptom screening for tuberculosis at each antenatal visit, and ensure that all women who screen positive are investigated for tuberculosis. This type of holistic approach is a simple intervention, but one that can potentially save women’s lives. A consistent feature amongst the big five causes of death in all the NCCEMD reports was that hypertensive disorders were the second commonest cause of death. Moreover, younger women seem to be at an increased risk of dying from complications due to pregnancy induced hypertensive disorders. In this study, about 16% of teenagers below 19 years died from hypertensive disorders. This calls for further enquiry into the causes of this increase in hypertensive disorders among these young women. Post-partum haemorrhage, puerperal sepsis, other maternal diseases and hypertensive disorders were the only four direct causes of death in four of the provinces. Conversely, the Eastern Cape, Free State, North West, Mpumalanga and Limpopo had a higher percentage of deaths from direct causes.

This study also found that a large proportion of deaths occurred in a health care facility. This proportion may include women who arrive in an already moribund state, those with complications and those who develop serious complications during a normal delivery and die without having received emergency care. Some studies have shown that delays in recognition and treatment of life-threatening complications, as well as sub-standard practises contribute directly to maternal deaths [5,6,11,27]. Vital registration data do not indicate the level of care of the hospitals in which the maternal deaths take place. However, the NCCEMD reports [2-6] indicate that most deaths occur at Level 1 and Level 2 institutions (Community health centres and district hospitals, and Regional Hospitals respectively). These are lower level institutions compared to the Level 3 institutions which are the provincial tertiary and national central hospitals and might have more qualified medical and health care personnel. The poorer provinces of South Africa (NW, NC, EC and MP) only have Level 1 and 2 institutions [2]. The high number of deaths at a health care facility could also be an indication of the improved use and attendance of health care facilities either during pregnancy or delivery.

About 10% of maternal deaths had an unknown place of death. These findings are in concurrence with the results of maternal mortality analysis by Statistics South Africa and WHO [1,19]. Documentation or non-documentation of place of death might influence the correct identification of maternal deaths leaving a gap in the identification of strategies to address maternal deaths.

The majority of maternal deaths were in the 25–29 age group. Substantial variations were found in the causes of death by age. Contributory causes included hypertensive disorders including eclampsia, HIV, respiratory tuberculosis, pneumonia, post-partum haemorrhage and puerperal sepsis. Studies conducted in South Africa, Pakistan and United Kingdom emphasize the importance of early and correct diagnosis and expertise in management so that maternal and perinatal morbidity can be reversed through early reference to tertiary care level hospitals [5,14,28].

The moderate to very high adjusted odds ratios found in the indirect causes compared to the weak to high adjusted odds ratios in the direct causes underscores the importance of immediate target specific interventions in lowering maternal deaths. A peculiar and surprising pattern in accidental injuries and neoplasms emerged. Although the number of deaths from neoplasms increased with age the risk of dying from neoplasms was higher among the younger women and lower among the older women and the Eastern Cape women had the highest risk of dying from neoplasms. The risk of dying from accidental injuries (including accidents, murders or suicides while a woman is pregnant) increased with age. Curiously, almost 70% of accidental injury deaths occurred in women below 29 years and Mpumalanga women had the highest accidental injury risk.

Ronsmans et al. [29] reported that earlier studies conducted in developed countries suggest that suicide might be triggered by pregnancy while some accidents might be pregnancy-related. An unpublished retrospective study by Khan [30] of a quantitative and qualitative review of all pregnancy related maternal deaths admitted to the Salt River Forensic Pathology Laboratory in Cape Town, South Africa between 1 January 2008 and 31 December 2012 highlights the violent homicidal death in pregnancy and the prevalence of intimate partner violence in pregnancy (https://open.uct.ac.za/handle/11427/6662). The study has brought to the attention of the public health community the importance of categorizing maternal deaths from suicide, homicide and accidental causes. It calls for the introduction of prevention and educational programmes into the antenatal programme in South Africa. The increase of neoplasm with age also highlights the importance of screening for cancer at very early stages of pregnancy in order to isolate pregnancy related cancers and existing cancers. It is also important to note that the risk of dying from HIV was significantly high for all the provinces, however, this risk was reduced at age-group levels. This may be attributable to the effect of a number of interventions targeted at halting transmission of HIV and the spread of AIDS, the medical male circumcision campaigns, as well as an increased number of people receiving ARVs [1,31]. Interestingly, pneumonia which is an opportunistic infection in HIV-positive people had a significantly reduced risk in all the provinces. The risk of dying from viral diseases was highest in Limpopo. These findings highlight the importance of advanced studies in maternal causes of death at provincial level.

## Conclusions

The major role played by indirect causes in this study makes the analysis of MMR using the demographic definition of maternal mortality difficult. The main aim of MMR is to target direct causes in order to improve the safety of pregnancies and deliveries. Trends in MMR in South Africa are increasingly reflecting trends in indirect causes. In addition, this study found that infectious diseases accounted for a higher proportion of maternal deaths than the direct obstetric causes. Indirect causes of death in pregnancy in developed countries are mostly comprised of chronic diseases, heart diseases and autoimmune diseases. Conversely, indirect causes in developing countries including South Africa mostly comprise infectious diseases which might be an indication of a generally poor health condition of the population in the country. These findings show that there is a potential to greatly reduce maternal mortality in South Africa, not only through the improved performance of health services for obstetric complications, but also by implementing measures to adequately prevent and treat a specific group of infectious diseases. Further research is recommended to establish the specific reasons for the high variations in the maternal causes of death at provincial level. The provincial variations in the leading causes of death indicate the importance of targeted interventions at provincial, district and local level.

Data generated at the country level provides an important framework; however, information at the local and regional level remains invaluable as it offers a perspective that highlights existing differences and gaps at the population level, thus leading to the identification of best practices and innovation opportunities. The results of this study provide strong support for prioritization of strategies as well as benchmarks for monitoring the impact of efforts to improve maternal health and reduce health inequalities at provincial level. The study also indicates that intervention should be both cause-specific and target-specific in combating the high maternal mortality in South Africa. Furthermore, the separation of indirect maternal death from direct maternal deaths in the calculation of the MMR is important, especially given the fact that the indirect causes are comprised of infectious diseases, has implications for intervention strategies. This is especially relevant where for example resources might be misdirected away from primary and secondary prevention strategies, which could have a huge effect on maternal mortality especially in a country with high HIV/AIDS related deaths like South Africa.

It is also important to develop, as a matter of urgency, policies that address poverty and reduce the growing economic and health inequality in South Africa (1). The absence of appropriate data for an analysis of the influence of social factors is a major limitation of this study.

## Endnote

^a^More detailed tables can be supplied upon request.

## Additional files

Additional file 1: Table S1 and Table S2. Variations in the direct and indirect causes of maternal deaths by socio-demographic characteristics. Additional file 2: Table S3 and Table S4. Adjusted Odds Ratio (OR) and 95% Confidence Interval (CI) of direct and indirect maternal mortality causes by socio-demographic characteristics.
