# Supplementary material for: A cross-sectional analysis of the geographic distribution and causes of maternal mortality in South Africa: 2002–2006
Source: BMC Public Health. 2015 Mar 19;15:273. doi: 10.1186/s12889-015-1597-5 (PMC4369832; doi:10.1186/s12889-015-1597-5)
Supplement: Additional file 1: Table S1 and Table S2. — Variations in the direct and indirect causes of maternal deaths by socio-demographic characteristics. [file 12889_2015_1597_MOESM1_ESM.docx]

**Additional file**

**Table S1 Variations in the indirect causes of maternal deaths by socio-demographic characteristics**

| Variables | Diarrhoea & Gastro Enteritis  n (%) | Tuberculosis  n (%) | Viral diseases  n (%) | HIV and related  n (%) | Pneumonia  n (%) | Protozoal diseases  n (%) | Other ill-defined  n (%) | Neoplasms  n (%) | Heart diseases  n (%) | Accidental injury  n (%) | Miscellaneous indirect  n (%) |
| --- | --- | --- | --- | --- | --- | --- | --- | --- | --- | --- | --- |
| Age group (years) |  |  |  |  |  |  |  |  |  |  |  |
| 10-19 | 9 (4.2) | 41 (4.5) | 18 (4.8) | 38(4.3) | 34(5.8) | 5(3.6) | 17(6.1) | 10(6.6) | 31(8.7) | 93(21.7) | 108(8.5) |
| 20-24 | 27 (12.6) | 160 (17.6) | 70 (18.5) | 185(20.9) | 102(17.5) | 24(17.5) | 57(20.6) | 17(11.2) | 60(16.8) | 106(24.8) | 229(18.0) |
| 25-29 | 60 (28.0) | 258 )28.4) | 127(33.6) | 250(28.2) | 168(28.8) | 42(30.7) | 56(20.2) | 22(14.5) | 60(16.8) | 92(21.5) | 283(22.3) |
| 30-34 | 54 (25.2) | 247 (27.2) | 75 (19.8) | 243(27.5) | 138(23.7) | 36(26.3) | 72(26.0) | 21(13.8) | 84(23.6) | 74(17.3) | 299(23.6) |
| 35-39 | 40 (18.7) | 112 (12.3) | 59 (15.6) | 104(11.8) | 76(13.0) | 24(17.5) | 33(11.9) | 23(15.1) | 70(19.7) | 34(7.9) | 17113.5) |
| 40-44 | 14 (6.5) | 56 (6.2) | 19 (5.0) | 41(4.6) | 42(7.2) | 6(4.4) | 23(8.3) | 27(17.8) | 32(8.9) | 15(3.5) | 95(7.5) |
| 45-49 | 6 (2.8) | 29 ( 3.2) | 8 (2.1) | 15(1.7) | 18(3.1) | 0(0) | 9(3.2) | 29(19.0) | 13(3.7) | 10(2.3) | 62(4.9) |
| 50+ | 4 (2.0) | 6(0.6) | 2 (0.5) | 8(0.9) | 5(.8) | 0(0) | 10(3.6) | 7(4.6) | 6(1.7) | 4(.9) | 22(1.7) |
| Total | **214 (100)** | **909 (100)** | **378(100)** | **884(100)** | **583(100)** | **137(100)** | **277(100)** | **152(100)** | **356(100)** | **428(100)** | **1269(100)** |
| Province of death |  |  |  |  |  |  |  |  |  |  |  |
| Western Cape | 4 (2.0) | 43 (4.7) | 17(4.5) | 75(8.5) | 14(2.4) | 3(2.2) | 11(4.0) | 13(8.6) | 20(5.6) | 37(8.6) | 62(4.9) |
| Eastern Cape | 26 (12.1) | 131 (14.4) | 50(13.2) | 125(14.1) | 71(12.2) | 13(9.5) | 53(19.1) | 14(9.2) | 39(10.9) | 70(16.9) | 181(14.3) |
| Northern Cape | 2 (0.9) | 24 (2.6) | 6(1.6) | 17(1.9) | 12(2.1) | 2(1.5) | 5(1.8) | 0(0) | 5(1.4) | 17(4.0) | 24(1.9) |
| Free State | 18 (8.4) | 57 (6.3) | 26(6.9) | 81(9.2) | 57(9.8) | 17(12.4) | 16(5.8) | 12(7.9) | 41(11.5) | 26(6.1) | 90(7.1) |
| KwaZulu-Natal | 59 (27.6) | 307 (33.8) | 113(29.9) | 233(26.3) | 125(21.3) | 47(34.3) | 77(27.8) | 30(19.7) | 70(19.7) | 118(27.6) | 321(25.2) |
| North West | 18 (8.4) | 76 (8.4) | 20(5.3) | 66(7.5) | 50(8.6) | 8(5.8) | 26(9.4) | 8(5.3) | 18(5.1) | 30(7.0) | 89(7.0) |
| Gauteng | 31 (14.5) | 144 (15.8) | 103(27.2) | 172(19.5) | 141(24.2) | 27(19.7) | 61(22.0) | 44(28.9) | 97(27.2) | 67(15.6) | 231(18.2) |
| Mpumalanga | 30 (14.0) | 70 (7.7) | 31(8.2) | 70(7.9) | 69(11.8) | 11(8.0) | 16(5.8) | 11(7.2) | 40(11.2) | 18(4.2) | 152(12.0) |
| Limpopo | 26 (12.1) | 57(6.3) | 12(3.2) | 45(5.1) | 44(7.5) | 9(6.6) | 12(4.3) | 20(13.2) | 26(7.3) | 45(10.5) | 119(9.4) |
| Total | **214 (100** | **909 (100)** | **378 (100)** | **884(100)** | **583(100)** | **137(100)** | **277(100)** | **152(100)** | **356(100)** | **428(100)** | **1269(100)** |
| Place of death |  |  |  |  |  |  |  |  |  |  |  |
| Health care facility | 138 (64.5) | 731 (80.4) | 301(79.6) | 737(83.4) | 408(70.0) | 124(90.5) | 102(36.8) | 113(74.3) | 260(73.0) | 175(40.5) | 930(73.3) |
| Home | 55 (25.7) | 95 (10.5) | 36(9.5) | 70(7.9) | 121(20.8) | 3(2.2) | 133(48.0) | 19(12.5) | 57(16.0) | 74(17.3) | 191(15.1) |
| Other | 21 (9.8) | 83 (9.1) | 41(0.8) | 77(8.7) | 54(9.2) | 10(7.3) | 42(15.2) | 20(13.2) | 39(11.0) | 179(41.8) | 148(11.0) |
| Total | 214 (100) | 909 (100) | 378(100) | 884(100) | 583(100) | 137(100) | 277(100) | 152(100) | 356(100) | 428(100) | 1269(100) |

**Table S2 Variations in the direct causes of maternal deaths by socio demographic characteristics**

| Variables | Abortion  n (%) | Hypertensive disorders  n (%) | Haemorrhage  n (%) | Sepsis  n (%) | Maternal infectious diseases  n (%) | Complications of labour  n (%) | Other maternal diseases  n (%) | Miscellaneous direct  n (%) |
| --- | --- | --- | --- | --- | --- | --- | --- | --- |
| Age group (years) |  |  |  |  |  |  |  |  |
| 10-19 | 25(5.6) | 127(15.9) | 34(8.7) | 40(12.8) | 18(6.7) | 26(13.8) | 30(7.7) | 36(9.0) |
| 20-24 | 91(20.6) | 183(22.9) | 69(17.7) | 80(25.5) | 55(14.1) | 38(20.2) | 72(18.5) | 66(16.5) |
| 25-29 | 118(26.7) | 197(24.6) | 78(20.0) | 81(25.9) | 91(33.8) | 40(21.3) | 112(28.9) | 104(26.1) |
| 30-34 | 130(29.5) | 148(18.5) | 95(24.4) | 58(18.5) | 61(22.7) | 41(21.8) | 101(26.0) | 109(27.3) |
| 35-39 | 58(13.2) | 96(12.0) | 76(19.5) | 43(13.7) | 33(12.3) | 28(14.9) | 48(12.4) | 57(14.3) |
| 40-44 | 18(4.1) | 45(5.6) | 34(8.7) | 11(3.5) | 9(3.3) | 13(6.9) | 18(4.6) | 18(4.5) |
| 45-49 | 1(0.2) | 2(0.3) | 3(0.8) | 0(0) | 2(0.7) | 2(1.1) | 3(0.8) | 7(1.8) |
| 50+ | 0(0) | 1(0.1) | 0(0) | 0(0) | 0(0) | 0(0) | 4(1.0) | 2(0.5) |
| Total | **441(100)** | **799(100)** | **389 (100)** | **313(100)** | **269 (100)** | **188 (100)** | **388 (100)** | **399 (100)** |
| Province of death |  |  |  |  |  |  |  |  |
| Western Cape | 15(3.4) | 37(4.6) | 9(2.3) | 5(1.6) | 11(4.1) | 10(5.3) | 12(3.1) | 17(4.3) |
| Eastern Cape | 66(15.0) | 130(16.3) | 77(19.8) | 61(19.5) | 48(17.8) | 36(19.1) | 59(15.2) | 52(13.0) |
| Northern Cape | 5(1.1) | 14(1.7) | 4(1.0) | 2(0.6) | 6(2.2) | 4(2.1) | 1(0.3) | 8(2.0) |
| Free State | 29(6.6) | 103(12.9) | 34(8.7) | 29(9.3) | 25(9.3) | 19(10.1) | 35(9.0) | 29(7.3) |
| KwaZulu-Natal | 98(22.2) | 176(22.0) | 81(20.8) | 94(30.3) | 70(26.0) | 47(25.0) | 71(18.3) | 84(21.0) |
| North West | 40(9.1) | 45(5.6) | 28(7.2) | 28(8.9) | 21(7.8) | 13(6.9) | 47(12.1) | 34(8.5) |
| Gauteng | 93(21.1) | 160(20.0) | 79(20.3) | 45(14.4) | 36(13.4) | 24(12.8) | 83(21.4) | 93(23.3) |
| Mpumalanga | 48(10.9) | 62(7.8) | 44(11.3) | 20(6.4) | 31(11.5) | 20(10.6) | 51(13.1) | 45(11.3) |
| Limpopo | 47(10.7) | 72(9.0) | 33(8.5) | 29(9.2) | 21(7.8) | 15(7.9) | 29(7.5) | 37(9.3) |
| Total | **441(100)** | **799(100)** | **389 (100)** | **313(100)** | **269 (100)** | **188 (100)** | **388 (100)** | **399 (100)** |
| Place of death |  |  |  |  |  |  |  |  |
| Health care facility | 339(76.9) | 634(79.3) | 304(78.1) | 241(77.0) | 225(83.6) | 137(72.8) | 281(72.4) | 289(72.4) |
| Home | 49(11.1) | 69(8.6) | 33(8.5) | 48(15.3) | 24(8.9) | 21(11.2) | 59(15.2) | 51(12.8) |
| Other | 53(12.0) | 96(12.0) | 52(13.4) | 24(7.7) | 20(7.4) | 30(16.0) | 48(12.4) | 59(14.8) |
| Total | 441(100) | 799(100) | 389 (100) | 313(100) | 269 (100) | 188 (100) | 388 (100) | 399 (100) |
