# Supplementary material for: A cross-sectional analysis of the geographic distribution and causes of maternal mortality in South Africa: 2002–2006
Source: BMC Public Health. 2015 Mar 19;15:273. doi: 10.1186/s12889-015-1597-5 (PMC4369832; doi:10.1186/s12889-015-1597-5)
Supplement: Additional file 2: Table S3 and Table S4. — Adjusted Odds Ratio (OR) and 95% Confidence Interval (CI) of direct and indirect maternal mortality causes by socio-demographic characteristics. [file 12889_2015_1597_MOESM2_ESM.docx]

**Additional file 2**

**Table S3: Adjusted Odds Ratio (OR) and 95% Confidence Interval (CI) of indirect maternal mortality causes by socio-demographic characteristics**

| Variables | Diarrhoea & Gastro Enteritis  OR (95%CI) | Tuberculosis  OR (95%CI) | Viral diseases  OR (95%CI) | HIV and related  OR (95%CI) | Pneumonia  OR (95%CI) | Protozoal diseases  OR (95%CI) | Other ill-defined  OR (95%CI) | Neoplasms  OR (95%CI) | Heart diseases  OR (95%CI) | Accidental injury  OR (95%CI) | Miscellaneous indirect  OR (95%CI) |  |
| --- | --- | --- | --- | --- | --- | --- | --- | --- | --- | --- | --- | --- |
| Age group (years) |  |  |  |  |  |  |  |  |  |  |  |  |
| 10-19 | 1(Ref) | 1(Ref) | 1(Ref) | 1(Ref) | 1(Ref) | 1(Ref) | 1(Ref) | 1(Ref) | 1(Ref) | 1(Ref) | 1(Ref) |  |
| 20-24 | 0.8(0.3-1.8) | 0.6(0.4-0.9)* | 0.6(0.3-1.1) | 0.5(0.3-0.7)*** | 0.8(0.5-1.3) | 0.5(0.2-1.4) | 0.6(0.3-1.1) | 1.5(0.7-3.4) | 1.4(0.9-2.2) | 2.3(1.6-3.2) | 1.3(1.0-1.7)* |  |
| 25-29 | 0.5(0.2-1.0) | 0.5(0.3-0.7)*** | 0.4(0.2-0.8)** | 0.5(0.3-0.7)*** | 0.7(0.4-1.0) | 0.4(0.1-1.1) | 0.9(0.5-1.6) | 1.6(0.7-3.6) | 2.0(1.2-3.1)** | 3.7(2.6-5.2) | 1.4(1.1-1.9)** |  |
| 30-34 | 0.5(0.2-1.0) | 0.5(0.3-0.7)*** | 0.8(0.4-1.3) | 0.4(0.3-0.7)*** | 0.8(0.5-1.2) | 0.4(0.1-1.2) | 0.6(0.3-1.2) | 1.6(0.7-3.6) | 1.3(0.8-2.0) | 4.5(3.2-6.4) | 1.2(0.9-1.6)* |  |
| 35-39 | 0.4(0.1-0.8)* | 0.6(0.4-0.9)** | 0.5(0.3-0.9)* | 0.6(0.4-0.9)* | 0.8(0.5-1.3) | 0.3(0.1-1.0)* | 0.9(0.5-1.7) | 0.8(0.3-1.7) | 0.8(0.5-1.3) | 5.9(3.8-9.1) | 1.2(0.9-1.6) |  |
| 40-44 | 0.5(0.2-1.3) | 0.6(0.3-0.9)* | 0.8(0.4-1.6)) | 0.8(0.5-1.3) | 0.7(0.4-1.2) | 0.7(0.2-2.4) | 0.6(0.3-1.3) | 0.3(0.1-0.7)** | 0.9(0.5-1.5) | 7.2(4.0-13.0) | 1.0(0.7-1.4) |  |
| 45+ | 0.6(0.2-1.6) | 0.6(0.4-1.1) | 1.1(0.5-2.5) | 1.0(0.5-1.7) | 1.0(0.6-1.9) | 0 | 0.8(0.4-1.6) | 0.1(0.0-0.3) | 1.1(0.6-2.1) | 6.2(3.3-11.4) | 0.7(0.5-1.1) |  |
| Province of death |  |  |  |  |  |  |  |  |  |  |  |  |
| Western Cape | 1(Ref) | 1(Ref) | 1(Ref) | 1(Ref) | 1(Ref) | 1(Ref) | 1(Ref) | 1(Ref) | 1(Ref) | 1(Ref) | 1(Ref) |  |
| Eastern Cape | 0.4(0.1-1.1) | 0.7(0.5-1.1) | 0.8(0.4-1.4) | 1.6(1.2-2.3)** | 0.4(0.2-0.9)* | 0.5(0.1-1.9) | 0.6(0.3-1.2) | 2.4(1.1-5.2)* | 1.3(0.7-2.3) | 1.6(1.0-2.5)* | 0.8(0.6-1.1) |  |
| Northern Cape | 0.7(0.1-4.2) | 0.6(0.3-1.0) | 1.1(0.4-2.8) | 1.9(1.0-3.4)* | 0.4(0.1-0.9)* | 0.5(0.0-3.2) | 1.0(0.3-3.0) | 0 | 1.5(0.5-4.1) | 0.7(0.3-1.4) | 0.9(0.5-1.6) |  |
| Free State | 0.3(0.1-1.0) | 1.1(0.7-1.6) | 0.9(0.5-1.7) | 1.4(1.0-2.0)* | 0.3(0.1-0.6)*** | 0.2(0.0-0.7)* | 1.4(0.6-3.1) | 1.5(0.6-3.5) | 0.7(0.4-1.2) | 1.9(1.1-3.3)* | 1.0(0.7-1.4) |  |
| KwaZulu-Natal | 0.3(0.1-0.9)* | 0.6(0.4-0.9)* | 0.7(0.4-1.2) | 1.8(1.3-2.5)* | 0.5(0.3-0.9)* | 0.3(0.0-1.0)* | 0.7(0.3-1.4) | 2.0(1.0-4.0)* | 1.4(0.8-2.4) | 1.5(1.0-2.4)* | 0.9(0.7-1.2) |  |
| North West | 0.3(0.1-0.9)* | 0.6(0.4-1.0) | 1.1(0.5-2.2) | 1.6(1.1-2.3)* | 0.3(0.2-0.6)** | 0.4(0.1-1.7) | 0.7(0.3-1.5) | 2.1(0.8-5.3) | 1.5(0.7-2.9) | 2.1(1.2-3.6)** | 0.9(0.6-1.3) |  |
| Gauteng | 0.4(0.1-1.3) | 1.1(0.7-1.6) | 0.5(0.3-1.0)* | 1.8(1.3-2.5)* | 0.3(0.1-0.5)*** | 0.4(0.1-1.3) | 0.6(0.3-1.2) | 1.0(0.5-2.0) | 0.7(0.4-1.1) | 2.5(1.6-4.1)* | 0.9(0.7-1.3) |  |
| Mpumalanga | 0.2(0.0-0.6)** | 1.0(0.6-1.5) | 0.9(0.5-1.7) | 2.0(1.4-3.0)* | 0.3(0.1-0.6)*** | 0.4(0.1-1.5) | 1.7(0.7-3.8) | 1.8(0.8-4.2) | 0.8(0.4-1.4) | 4.1(2.2-7.6) | 0.6(0.4-0.8)** |  |
| Limpopo | 0.2(0.0-0.6)** | 1.0(0.6-1.5) | 1.9(0.9-4.2) | 2.6(1.7-4.0) | 0.4(0.2-0.8)** | 0.4(0.1-1.5) | 1.6(0.6-3.8) | 0.8(0.4-1.8) | 1.0(0.5-1.9) | 1.1(0.7-1.9)* | 0.6(0.4-0.9)* |  |
| Place of death |  |  |  |  |  |  |  |  |  |  |  |  |
| *Health care facility* | 1(Ref) | 1(Ref) | 1(Ref) | 1(Ref) | 1(Ref) | 1(Ref) | 1(Ref) | 1(Ref) | 1(Ref) | 1(Ref) | 1(Ref) |  |
| Home | 0.5(0.3-0.7)** | 1.7(1.3-2.1)  1.6(1.2-2.0)** | 1.6(1.1-2.3)**  1.3(0.9-1.8) | 2.3(1.8-3.0)  1.7(1.3-2.3) | 0.6(0.5-0.8)***  1.3(1.0-1.8)* | 8.6(2.7-27.3)***  1.9(1.0-3.8)* | 0.1(0.0-0.1)  0.4(0.2-0.6) | 1.6(0.9-2.7)*  1.1(0.7-1.9) | 1.0(0.7-1.3)  1.2(0.8-1.7) | 0.4(0.3-0.5)  0.1(0.1-0.1) | 1.1(0.9-1.3)  1.1(0.9-1.4) |  |
| Other | 1.0(0.6-1.7) |  |  |  |  |  |  |  |  |  |  |  |

**Table S4: Adjusted Odds Ratio (OR) and 95% Confidence Interval (CI) of direct maternal mortality causes by socio-demographic characteristics**

| Variables | Abortion  OR (95%CI) | Hypertensive disorders  OR (95%CI) | Haemorrhage  OR (95%CI) | Sepsis  OR (95%CI) | Maternal infectious diseases  OR (95%CI) | Complications of labour  OR (95%CI) | Other maternal diseases  OR (95%CI) | Miscellaneous direct  OR (95%CI) |  |  |  |
| --- | --- | --- | --- | --- | --- | --- | --- | --- | --- | --- | --- |
| Age group (years) |  |  |  |  |  |  |  |  |  |  |  |
| 10-19 | 1(Ref) | 1(Ref) | 1(Ref) | 1(Ref) | 1(Ref) | 1(Ref) | 1(Ref) | 1(Ref) |  |  |  |
| 20-24 | 1.9(1.2-3.1)** | 0.6(0.4-0.8)** | 1.0(0.7-1.6) | 1.0(0.6-1.5) | 1.6(0.9-2.8) | 0.7(0.4-1.2) | 1.2(0.7-1.8) | 0.9(0.5-1.4) |  |  |  |
| 25-29 | 2.0(1.3-3.2)** | 0.5(0.3-0.6) | 0.9(0.6-1.4) | 0.8(0.5-1.2) | 2.3(1.3-3.9)** | 0.6(0.3-1.0) | 1.5(0.9-2.3)* | 1.1(0.7-1.7) |  |  |  |
| 30-34 | 2.5(1.6-4.0)* | 0.4(0.3-0.5) | 1.3(0.8-2.0) | 0.6(0.4-0.9)* | 1.6(0.9-2.9)* | 0.7(0.4-1.2) | 1.4(0.9-2.3) | 1.3(0.9-2.0) |  |  |  |
| 35-39 | 1.8(1.1-3.0)* | 0.4(0.3-0.6) | 1.9(1.2-2.9)** | 0.8(0.5-1.3) | 1.5(0.8-2.8) | 0.8(0.4-1.4) | 1.1(0.7-1.8) | 1.1(0.7-1.8) |  |  |  |
| 40-44 | 1.4(0.7-2.7) | 0.6(0.4-0.9)* | 2.3(1.3-3.9)** | 0.5(0.2-1.0) | 1.0(0.4-2.4) | 1.0(0.5-2.0) | 1.1(0.6-2.1) | 0.9(0.5-1.7) |  |  |  |
| 45+ | 0.4(0.0-3.6) | 0.2(0.0-0.7)* | 1.1(0.3-4.0) | 0 | 1.3(0.2-6.1) | 0.8(0.1-4.0) | 3.3(1.2-8.5)* | 4.1(1.7-9.9)** |  |  |  |
| Province of death |  |  |  |  |  |  |  |  |  |  |  |
| Western Cape | 1(Ref) | 1(Ref) | 1(Ref) | 1(Ref) | 1(Ref) | 1(Ref) | 1(Ref) | 1(Ref) |  |  |  |
| Eastern Cape | 0.9(0.5-1.8) | 0.6(0.4-1.0) | 2.0(0.9-4.2)* | 2.8(1.1-7.2)* | 1.0(0.5-2.0) | 0.7(0.3-1.5) | 1.1(0.512.1) | 0.6(0.3-1.1) |  |  |  |
| Northern Cape | 0.8(0.2-2.5) | 0.9(0.4-2.0) | 1.3(0.3-4.5) | 0.9(0.1-4.9) | 1.6(0.5-4.7) | 1.0(0.3-3.5) | 0.1(0.0-1.5) | 1.3(0.5-3.3) |  |  |  |
| Free State | 0.7(0.3-1.3) | 1.0(0.6-1.7) | 1.5(0.7-3.3) | 2.1(0.8-5.7) | 0.8(0.4-1.8) | 0.7(0.3-1.5) | 1.1(0.5-2.2) | 0.6(0.3-1.2) |  |  |  |
| KwaZulu-Natal | 1.0(0.5-1.9) | 0.6(0.4-1.0)* | 1.5(0.7-3.2) | 3.1(1.2-7.9)* | 1.0(0.5-2.0) | 0.7(0.3-1.5) | 0.9(0.4-1.8) | 0.7(0.4-1.3) |  |  |  |
| North West | 1.2(0.6-2.3) | 0.4(0.2-0.7)** | 1.5(0.6-3.3) | 2.7(1.0-7.3)* | 0.9(0.4-2.0) | 0.5(0.2-1.2) | 1.8(0.9-3.7) | 0.8(0.4-1.5) |  |  |  |
| Gauteng | 1.1(0.6-2.1) | 0.7(0.4-1.1) | 1.7(0.8-3.5) | 1.8(0.6-4.6) | 0.5(0.2-1.2) | 0.4(0.1-0.9)* | 1.3(0.7-2.6) | 1.0(0.5-1.8) |  |  |  |
| Mpumalanga | 1.1(0.6-2.2) | 0.5(0.3-0.8)** | 2.0(0.9-4.2) | 1.3(0.4-3.6) | 1.0(0.5-2.2) | 0.6(0.3-1.5) | 1.5(0.7-3.0) | 0.9(0.5-1.7) |  |  |  |
| Limpopo | 1.3(0.7-2.4) | 0.7(0.4-1.1) | 1.5(0.7-3.3) | 2.4(0.9-6.5) | 0.7(0.3-1.7) | 0.5(0.2-1.3) | 1.0(0.4-2.0) | 0.8(0.4-1.6) |  |  |  |
| Place of death |  |  |  |  |  |  |  |  |  |  |  |
| Health care facility | 1(Ref) | 1(Ref) | 1(Ref) | 1(Ref) | 1(Ref) | 1(Ref) | 1(Ref) | 1(Ref) | 1(Ref) | 1(Ref) | 1(Ref) |
| Home | 0.9(0.6-1.3) | 0.7(0.5-1.0) | 0.6(0.4-1.0) | 1.5(1.0-2.1)* | 0.6(0.4-1.0) | 1.0(0.6-1.7) | 1.4(1.0-1.9)* | 1.2(0.9-1.7) |  |  |  |
| Other | 0.9(0.6-1.3) | 1.0(0.7-1.3) | 1.0(0.7-1.4) | 0.6(0.4-0.9)* | 0.5(0.3-0.9)* | 1.5(1.0-2.3)* | 1.0(0.7-1.4) | 1.3(0.9-1.8) |  |  |  |

0 – no data

*** P< .001

** P< 0.01

* P<.05
